# Inflammo-immune perspective on the association of eight migraine risk factors with migraine: a multi-omics Mendelian randomization study

**DOI:** 10.3389/fneur.2024.1440995

**Published:** 2024-08-07

**Authors:** Jiaxi Zhao, Rong Chen, Mengqi Luo, Hongping Gong, Kaixin Li, Qian Zhao

**Affiliations:** ^1^General Practice Ward/International Medical Center Ward, General Practice Medical Center, West China Hospital, Sichuan University, Chengdu, Sichuan, China; ^2^Department of Rehabilitation Medicine, The First Affiliated Hospital, Sun Yat-sen University, Guangzhou, Guangdong, China; ^3^Department of Gastroenterology and Hepatology, West China Hospital, Sichuan University, Chengdu, Sichuan, China; ^4^Princess Alexandra Hospital, Metro South Hospital and Health Service, Brisbane, QLD, Australia; ^5^Centre for Health Services Research, The University of Queensland, Brisbane, QLD, Australia

**Keywords:** inflammatory proteins, immune cells, multiomics, migraine, Mendelian randomization

## Abstract

**Background:**

Migraine risk factors are associated with migraine susceptibility, yet their mechanisms are unclear. Evidence suggests a role for inflammatory proteins and immune cells in migraine pathogenesis. This study aimed to examine the inflammo-immune association between eight migraine risk factors and the disorder.

**Methods:**

This study utilized inverse variance weighted (IVW) method and colocalization analysis to explore potential causal relationships between eight migraine risk factors, migraine, 731 immune cells, and 91 circulating inflammatory proteins. Mediation Mendelian randomization (MR) was further used to confirm the mediating role of circulating inflammatory proteins and immune cells between the eight migraine risk factors and migraine.

**Results:**

Migraine risk factors are linked to 276 immune cells and inflammatory proteins, with cigarettes smoked per day strongly co-localized with CD33-HLA DR+ cells. Despite no co-localization, 23 immune cells/inflammatory proteins relate to migraine. Depression, all anxiety disorders, and sleep apnea are correlated with migraine, and all anxiety disorders are supported by strong co-localization evidence. However, the mediating effect of inflammatory proteins and immune cells between eight migraine risk factors and migraine has not been confirmed.

**Conclusion:**

We elucidate the potential causal relationships between eight migraine risk factors, migraine, immune cells, and inflammatory proteins, enhancing our understanding of the molecular etiology of migraine pathogenesis from an inflammatory-immune perspective.

## Introduction

Migraine, with its exact pathomechanism still unknown, impacts more than 1.1 billion individuals globally (1). Its extensive prevalence and associated disability yield a spectrum of adverse and significant consequences not only for those directly afflicted, but also for their families, and society (2, 3). In 2019, the worldwide age-standardized point prevalence and annual incidence rate of migraines stood at 14,107.3 and 1,142.5 per 100,000 individuals, reflecting a respective increment of 1.7 and 2.1% since 1990 (4). The economic ramifications of migraine are substantial, encompassing both direct healthcare expenses, such as medical consultations and medication costs, and indirect costs, including decreased productivity (5).

The pathogenesis of migraines is characterized by a multifaceted interplay of genetic predisposition, neurological dysregulation, and environmental influences (6). Abnormal neuronal activity precipitates the activation of the trigeminovascular system, instigating neurogenic inflammation and cerebral blood vessel vasodilation (7). The over 180 recognized variations are part of several intricate networks of “pro-migraine” molecular irregularities, predominantly of neuronal or vascular origin (8). Identifying key risk factors associated with migraine susceptibility is imperative for comprehensive management strategies (9, 10). Factors such as alcohol dependence (11, 12), smoking (13), depression (14, 15), anxiety (16), insomnia or sleep apnea (17), obesity (18), glucocorticoid use (19, 20) have been implicated in migraine pathogenesis. These risk factors may contribute to migraines by influencing neurotransmitter control, hormonal disruptions, and immune system irregularities (21). Notably, pertinent research has uncovered the intricate connections between migraines and inflammatory processes as well as immune system activation (22). Heightened levels of inflammatory cytokines, comprising IL-6, TNF-α, and CGRP, play a role in pain transmission and vascular alterations during migraine episodes (23). Furthermore, immune cells such as T cells and macrophages sustain an inflammatory milieu that intensifies migraine symptoms (24).

Understanding the association between migraine risk factors and inflammatory responses is crucial for elucidating migraine pathophysiology. While common risk factors like depression or anxiety (25), insomnia (26) and obesity (27, 28) have been implicated in modulating immune function and promoting inflammation. Epidemiological research has revealed a reciprocal association between migraines and these risk factors, wherein individuals with one condition are at an increased risk of developing the other (17, 29). This correlation is thought to stem from common genetic influences, disturbances in neurotransmitter function, inflammation, hormonal changes, and concurrent conditions present in both disorders (6). The precise mechanistic interactions remain subject to ongoing investigation. In the pursuit of clarifying the causative relationships between migraine risk factors and inflammatory responses, methodologies such as Mendelian randomization (MR) offer promising avenues (30). By leveraging genetic variations associated with exposure factors as instrumental variables, MR enables the exploration of causal associations while minimizing confounding effects (31).

In this study, we utilized extensive genome-wide association studies (GWAS) summary statistics to assess the interconnections among 91 circulating inflammatory proteins, 731 immune cells, and the occurrence of migraines triggered by eight specific factors. These factors encompass alcohol dependence, depression, anxiety disorders, insomnia, sleep apnea, obesity, glucocorticoid use, and the daily consumption of cigarettes. Using MR and colocalization analysis, we identified genetic links between migraine risk factors, inflammatory proteins, immune cells, and migraines. These factors may serve as novel biomarkers for early diagnosis, prevention, and monitoring of migraines.

## Methods

### Study design

**Figure 1:**
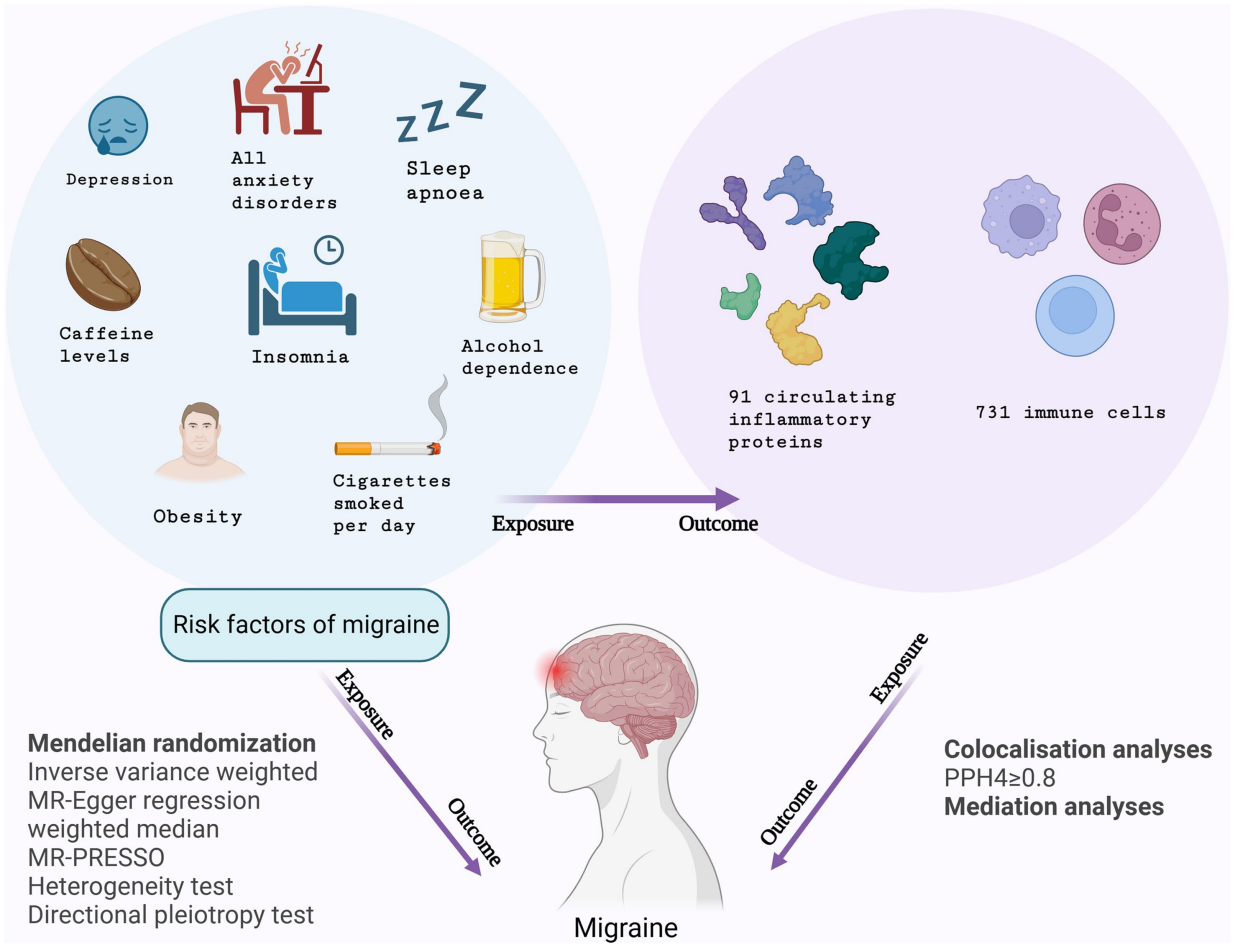
Study design overview.

This study conducted MR analyses to explore the inflammo-immune perspective concerning the correlation between eight migraine risk factors and the occurrence of migraines, utilizing GWAS data on 91 circulating inflammatory proteins and 731 immune cells. Initially, MR analyses were conducted with the eight migraine risk factors serving as exposures and circulating inflammatory proteins and immune cells as respective outcomes. Subsequently, inflammatory proteins and immune cells were considered as exposure factors, and the risk of migraines was the primary outcome. Following the completion of the study, additional MR analyses were carried out, with migraine risk factors identified as the exposures and migraines as the primary outcome. The study also involved a mediation analysis aiming to ascertain the mediation effects of circulating inflammatory proteins and immune cells on the risk of migraines associated with these risk factors. Figure 1 depicts the design of the study.

The MR analysis hinges on three critical assumptions: relevance, independence, and exclusion restriction. The first assumes a strong association between the instrumental and exposure variables. The second stipulates the independence of the instrumental variable from confounding factors. Lastly, the exclusion restriction assumption ensures that the instrumental variable affects the outcome solely through the exposure variable, with no direct relationship to the outcome (32).

GWAS summary statistics in this study originated from public databases. Ethical approvals and informed consent from participants have been obtained. No further ethical approval or consent required.

### Data sources of eight risk factors of migraine and migraine

In our study, we comprehensively included eight migraine risk factors, six of which were derived from the FinnGen R10 study and the remaining two from alternative databases (33). FinnGen, a public-private partnership leveraging Finnish genotype and digital health data, aims to decipher genetic variations and disease risks in an isolated population. Our research capitalized on the most up-to-date FinnGen data, encompassing alcohol dependence, depression, all anxiety disorders, insomnia, sleep apnea, and obesity. The number of cases ranged significantly, from 4,801 individuals with insomnia to 47,696 suffering from depression. The comprehensive GWAS summary statistics can be accessed and downloaded from: https://finngen.gitbook.io/documentation/data-download.

The GWAS conducted on glucocorticoid use included a comprehensive dataset of 178,726 participants, encompassing 13,102 cases and 165,624 controls (33), sourced from: https://www.nature.com/articles/s41588-021-00931-x. And we incorporated data on the number of cigarettes smoked per day from the Within Family GWAS Consortium, which included 24,784 European individuals.^1^ Similarly, the migraine data were derived from the FinnGen R10 study, encompassing 20,908 cases, accessible at: https://finngen.gitbook.io/documentation/data-download.

### Data sources of 91 circulating inflammatory proteins and 731 immune cells

The data on circulating inflammatory proteins were derived from a meta-analysis of 11 cohorts, encompassing 14,824 subjects of unrelated individuals of European ancestry (34). This is a manifestation of proteomics. The complete protein GWAS summary statistics are available for download at: https://www.phpc.cam.ac.uk/ceu/proteins and the EBIGWAS catalog (IDs: GCST90274758-GCST90274848). The SNP data utilized for 731 immune cell analysis were sourced from Valeria Orrù et al.’s study, encompassing 3,757 Sardinians (35). The statistics can be archived in the GWAS Catalog, with accession numbers ranging from GCST0001391^2^ to GCST0002121.^3^ Detailed measurement methods can be found in the original publication.

### Selection of instrumental variables

In the present study, the selection criteria of instrumental variable encompassed the following: (a) a genome-wide significant association with a *p*-value less than 5 × 10^−8^, (b) evidence of independent association linkage disequilibrium (LD) clustering, characterized by an r^2^ value below 0.001 and a distance of less than 10,000 kb, (c) a minor allele frequency (MAF) exceeding 0.01. All the data are openly accessible and exempt from ethical committee review. Furthermore, we assess the strength of the instrumental variable using the F-statistic, with >10 indicating strong association (F = R^2^(n-k-1)/k(1-R^2^), n = sample size, R^2^ was estimated by minor allele frequency (MAF) and β value: R^2^ = 2 × MAF × (1−MAF) × β^2^) (36).

### MR analyses

In this study, we conducted three MR analyses. Firstly, we performed an MR analysis (MR 1) using eight risk factors of migraine as the exposure factor and 91 circulating inflammatory proteins and 731 immune cells as the outcome measures. Secondly, we conducted another MR analysis (MR 2) with circulating inflammatory proteins and immune cells as the exposure factor and migraine as the outcome measure. Lastly, we performed the third MR analysis (MR 3) using eight risk factors of migraine as the exposure and migraine as the outcome.

Additionally, we validated whether circulating inflammatory proteins and immune cells serve as mediators between eight risk factors of migraine and migraine (36, 37). We primarily comprised two sequential steps. Firstly, we employed the MR approach, utilizing the eight risk factors of migraine as the exposure variables and migraine as the outcome variable, to elucidate the influence of these risk factors on the development of migraine. Subsequently, the circulating inflammatory proteins and immune cells were designated as the exposure variables, while GWAS of migraine served as the outcome. Ultimately, an intermediate analysis was conducted, combining the identified positive risk factors of migraine and the inflammatory proteins and immune cells, to investigate their mediating effects in the pathogenesis of migraine.

In assessing the causal link between exposures and outcomes, we primarily utilized the inverse-variance weighting (IVW) approach, with MR-Egger regression (38), weighted median, and MR pleiotropy residual sum and outlier (MR-PRESSO) test as supplementary ones (38). Cochran’s Q method detected heterogeneity when its *p*-value was <0.05 (39). By utilizing the intercept of the MR-Egger regression (40), we assessed whether directional pleiotropy has an impact on the causal estimation, and the MR-PRESSO test was used to correct pleiotropy effects by excluding outliers (41, 42). The false discovery rate (FDR) method was employed to correct for multiple comparisons, and the association was considered statistically significant if the *P*_FDR_ of the IVW method was less than 0.05 (43). Additionally, a *p*-value of less than 0.05 was deemed indicative of a potential association. The MR analysis was performed with the TwoSampleMR package (v0.5.6) in R software (v4.2.2).

### Colocalization analysis

Bayesian colocalization analyses was conducted to determine if the associations between the exposures and outcomes were motivated by linkage disequilibrium, considering five hypothetical scenarios: no association (Hypothesis 0), independent association of trait 1 (Hypothesis 1), independent association of trait 2 (Hypothesis 2), association between the two traits with different causal variations (Hypothesis 3), and association between the two traits with identical causal variations (Hypothesis 4). By calculating their respective posterior probabilities (PPH0 to PPH4), we were able to quantify the likelihood of each hypothesis. When the PPH4 is greater than 0.8, it was considered as strong support for the colocalization of the two traits (44). The “coloc” package used in the analysis was downloaded from: https://github.com/chr1swallace/coloc.

## Results

The summary data information were presented in Supplementary Table S1. During the power examination of all instrumental variables, the minimum F-statistic exceeded 10. The largest sample size was for depression, with 47,696 samples, while the smallest sample size was for 731 immune cells, with 3,757 samples, of which the statistical power is relatively low.

### Associations of genetic liability to eight migraine risk factors with 91 circulating inflammatory proteins and 731 immune cells

**Figure 2:**
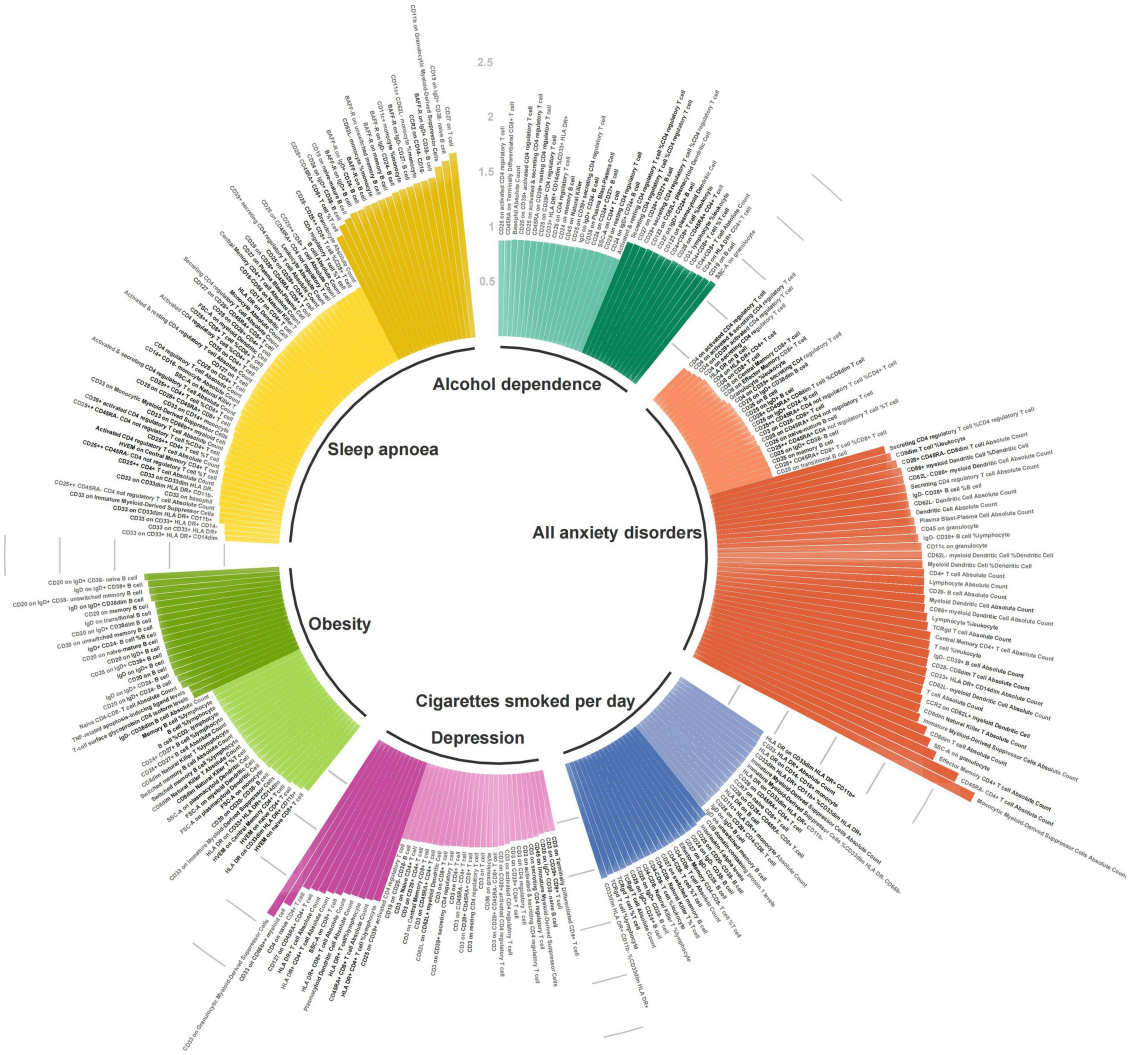
Associations of genetic liability to eight migraine risk factors with 91 circulating inflammatory proteins and 731 immune cells.

We performed MR analyses using eight migraine risk factors as exposures and 91 circulating inflammatory proteins and 731 immune cells as outcomes, respectively. Figure 2 and Supplementary Table S2 shows the summary of the analysis results. When circulating inflammatory proteins were used as the outcomes, the number of cigarettes smoked per day was a potentially positive regulatory factor for Interleukin-1-alpha levels (OR = 1.035, 95% CI 1.006–1.065, *p* = 0.017, *P*_FDR_ = 0.782) and CUB domain-containing protein 1 levels (OR = 1.030, 95% CI 1.005–1.055, *p* = 0.017, *P*_FDR_ = 0.782). Moreover, obesity was potentially positively correlated with TNF-related apoptosis-inducing ligand levels (OR = 1.066, 95% CI 1.004–1.131, *p* = 0.036, *P*_FDR_ = 0.695) and T-cell surface glycoprotein CD6 isoform levels (OR = 1.061, 95% CI 1.001–1.124, *p* = 0.044, *P*_FDR_ = 0.695). The results showed no heterogeneity and pleiotropy (*p*-value>0.05). However, no potential causal relationship was found for alcohol dependence, depression, anxiety disorders, insomnia, sleep apnoea, or glucocorticoid use with inflammatory proteins, in which the *p* values before and after FDR-corrected are all greater than 0.05.

With regard to immune cells, alcohol dependence, cigarettes smoked per day, depression, all anxiety disorders, sleep apnoea, and obesity were, respectively, associated with 33, 31, 38, 62, 69, and 39 types of immune cells, respectively. In the heterogeneity and pleiotropy analyses, both *p*-values exceeded the significance threshold of 0.05. Among the immune cells related to cigarettes smoked per day, CD33- HLA DR+ Absolute Count highly supported the colocalization analysis (PH4 = 0.814), while HLA DR on CD33dim HLA DR+ CD11b + (PH4 = 0.696) and HVEM on naive CD4+ T cell (PH4 = 0.532) moderately supported the colocalization analysis in relation to obesity (Supplementary Table S5).

### Associations of 91 circulating inflammatory proteins and 731 immune cells with the risk of migraine

**Figure 3:**
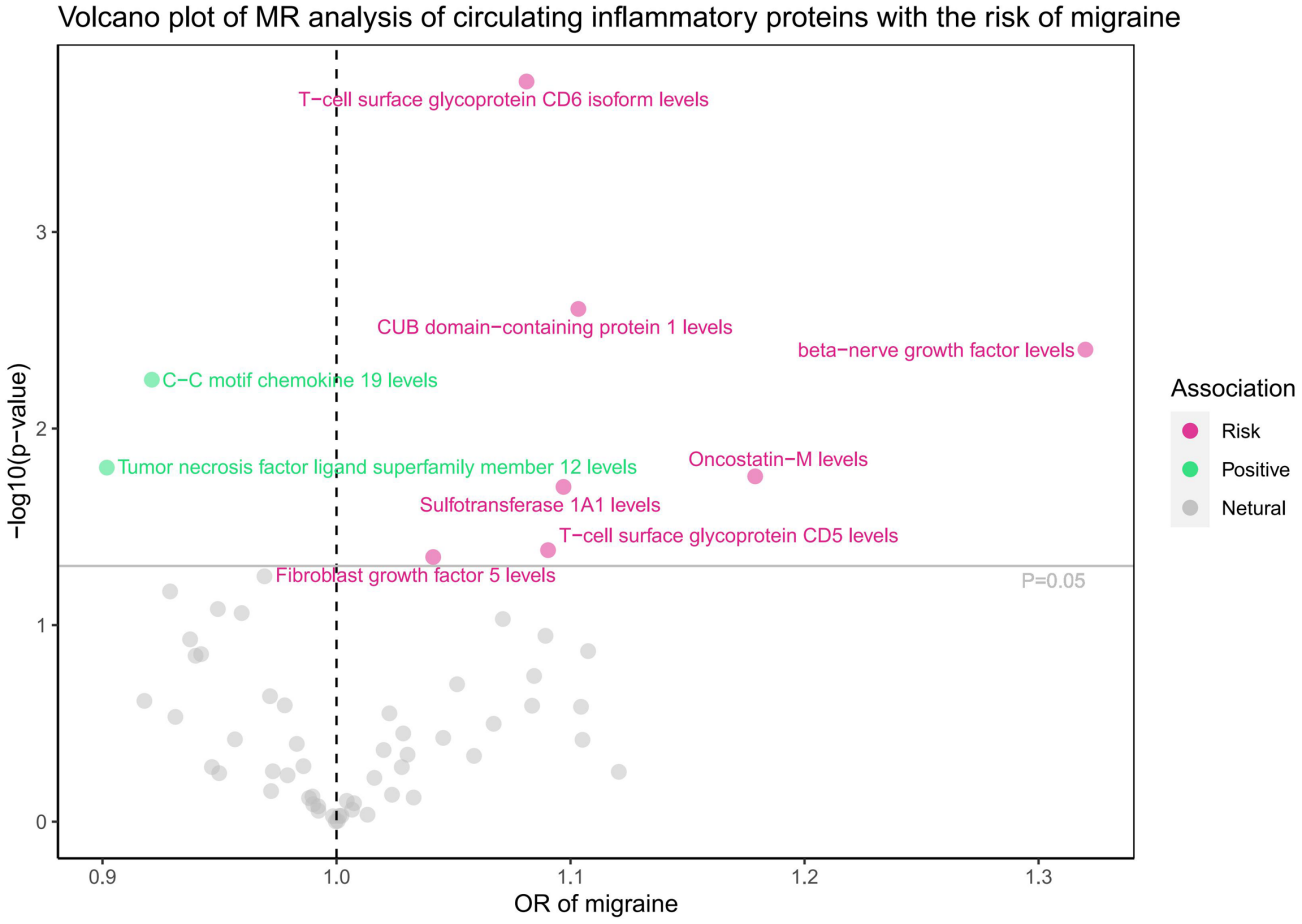
Associations of 91 circulating inflammatory proteins with the risk of migraine.

**Figure 4:**
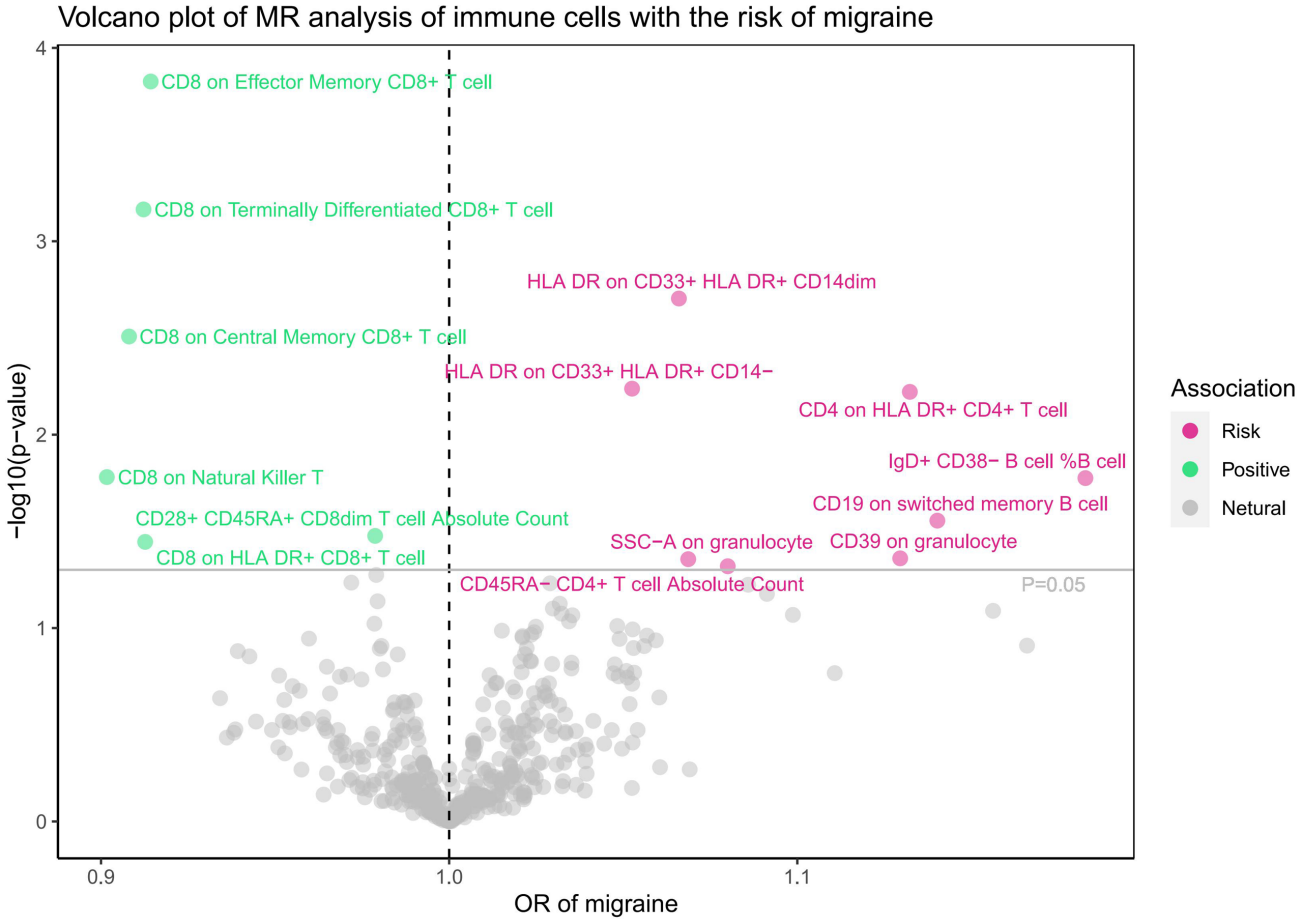
Associations of 731 immune cells with the risk of migraine.

Subsequently, we conducted a comprehensive MR analysis, with a focus on 91 circulating inflammatory proteins and 731 immune cells as the exposures, and the risk of migraine as the outcome. The results of this analysis are presented in Figures 3, 4 and Supplementary Table S3. Our findings demonstrated a clear causal relationship between nine circulating inflammatory proteins and the risk of migraine. The increased risk of migraine was potentially associated with seven inflammatory proteins: T-cell surface glycoprotein CD6 isoform levels (OR = 1.081, 95% CI 1.038–1.126, *p* = 0.000), CUB domain-containing protein 1 levels (OR = 1.103, 95% CI 1.035–1.176, *p* = 0.002), beta-nerve growth factor levels (OR = 1.320, 95% CI 1.093–1.594, *p* = 0.004), Oncostatin-M levels (OR = 1.179, 95% CI 1.029–1.350, *p* = 0.018), Sulfotransferase 1A1 levels (OR = 1.097, 95% CI 1.015–1.186, *p* = 0.020), T-cell surface glycoprotein CD5 levels (OR = 1.090, 95% CI 1.003–1.185, *p* = 0.042), and Fibroblast growth factor 5 levels (OR = 1.041, 95% CI 1.001–1.083, *p* = 0.045). Conversely, C-C motif chemokine 19 levels (OR = 0.921, 95% CI 0.869–0.976, *p* = 0.006) and Tumor necrosis factor ligand superfamily member 12 levels(OR = 0.902, 95% CI 0.829–0.981, *p* = 0.016) were associated with a reduced risk of migraine. After FDR correction, only the *p*-value for the isoform levels of T-cell surface glycoprotein CD6 is 0.01, while all other *p*-values are greater than 0.05, indicating a potential statistical significance. Furthermore, we uncovered a significant causal association between 14 specific immune cells and the development of migraine. Eight immune cells were positively correlated with the risk of migraine, while six types of immune cells were negatively correlated. However, all the *p*-values after FDR correction are >0.05. Utilizing IVW as our primary analytical method, we observed consistent directions in the effect size estimates when compared to alternative methods. This consistency strengthens the reliability of our results. Additionally, we did not reveal any evidence of heterogeneity or pleiotropy (*p*-value>0.05).

The analysis of T-cell surface glycoprotein CD6 isoform levels (PH4 = 0.614) and CD8 expression on terminally differentiated CD8+ T cells (PH4 = 0.533) provided moderate evidence in support of the colocalization analysis in the context of migraine (Supplementary Table S6).

### Associations of migraine risk factors with the risk of migraine

**Figure 5:**
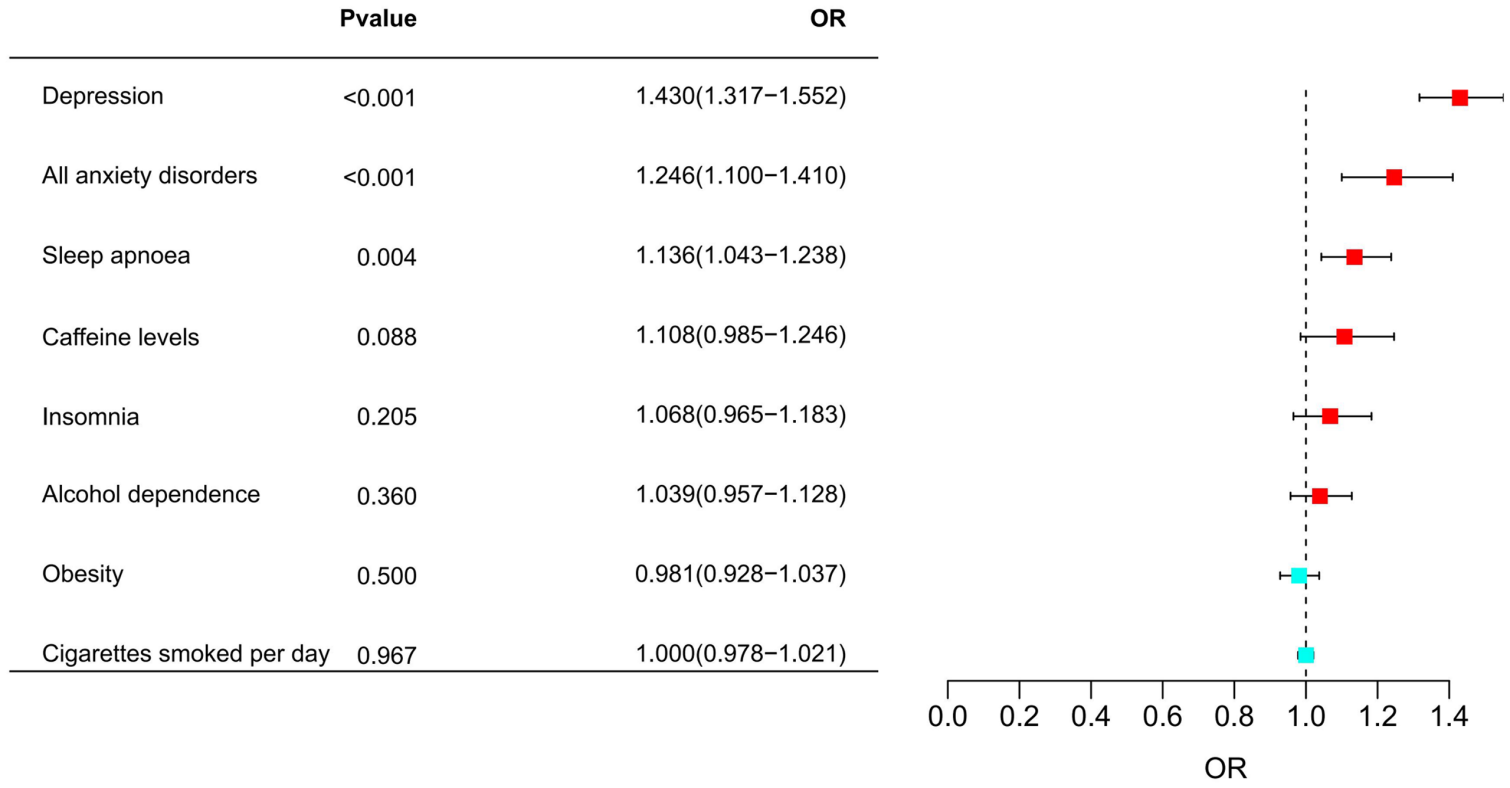
Associations of migraine risk factors with the risk of migraine.

Later, MR analyses were undertaken, wherein migraine risk factors were identified as the exposures, and migraine emerged as the outcome. Depression was associated with an increased risk of migraine (OR = 1.430, 95% CI 1.317–1.552, *p* = 1.165 × 10^−17^, *P*_FDR_ = 9.323 × 10^−17^). Additionally, all anxiety disorders and sleep apnoea were also positively correlated with an increased risk of migraine, with respective ORs of 1.246 (95% CI 1.1–1.41, *P*_FDR_ = 2.073 × 10^−3^) and 1.136 (95% CI 1.043–1.238, *p* = 3.579 × 10^−3^, *P*_FDR_ = 9.544 × 10^−3^). Among them, all anxiety disorders highly supported the colocalization analysis with migraine (PH4 = 0.896). The details were presented in Figure 5 and Supplementary Tables S4, S7.

### Mediation effects of 91 circulating inflammatory proteins and 731 immune cells on migraine risk factors – migraine risk

Despite a potential association between the risk factors and migraine, after conducting a mediation analysis that integrated positive migraine risk factors with circulating inflammatory proteins and immune cells, we discovered no indication that these proteins and cells mediate the risk of migraine (Supplementary Table S8).

## Discussion

In this study, we have discerned the associations among eight migraine risk factors, the 91 inflammatory proteins and 731 immune cells, and migraine. MR analysis has allowed us to explore the complex interplay between these factors in unprecedented detail. By examining the associations among these different factors, we aimed to shed light on the complex biological processes that underlie migraines. Our findings provide valuable insights into the complex interplay between these factors.

Our investigation has unveiled correlation between eight migraine risk factors and the presence of circulating inflammatory proteins and immune cells. Specifically, cigarettes smoked per day may served as a potential factor in elevating in Interleukin-1-alpha (IL-1α) and CUB domain-containing protein 1 (CDCP1). From previous study cigarette smoke exposure induces inflammation marked by rapid and sustained neutrophil infiltration, IL-1α, release and altered surfactant homeostasis (45). IL-1α is a pro-inflammatory cytokine that plays a crucial role in the initiation and propagation of inflammatory responses. Upregulation of IL-1α has been associated with various inflammatory diseases and is now implicated in the inflammatory response triggered by smoking. Increased levels of IL-1α can induce the recruitment and activation of additional immune cells, perpetuating a cycle of inflammation that could potentially contribute to the pathogenesis of migraines (46). CDCP1 is a transmembrane protein that has been implicated in cell adhesion and migration (47), may processes integral to the inflammatory response. We supposed that elevated levels of CDCP1 could potentially enhance the migratory capacity of immune cells, facilitating their infiltration into sites of inflammation (48). In the context of migraines, these changes induced by smoking could potentially exacerbate the neurogenic inflammation characteristic of the condition, leading to more frequent or severe attacks (49). However, further research is needed to fully elucidate the mechanistic link between cigarette smoke, IL-1α and CDCP1 elevation, and migraine pathogenesis.

Moreover, in the study, obesity was associated with altered levels of TNF-related apoptosis-inducing ligand (TRAIL) and T-cell surface glycoprotein CD6 isoform, aligning closely with findings from prior research. Obesity is a state of chronic low-grade inflammation, often referred to as “metaflammation.” This inflammatory state is characterized by alterations in various immune markers, including TRAIL and CD6. TRAIL is a cytokine known for its ability to induce apoptosis in cells (50). Funcke et al. found that TRAIL in regulating adipose tissue homeostasis by promoting the proliferation of tissue-resident precursor cells (51). In the context of obesity, TRAIL levels are often dysregulated. Combined with the result in this study, the dysregulation may contribute to the inflammatory state observed in obesity, and potentially exacerbate the inflammatory response seen in conditions such as migraines. CD6 is a surface glycoprotein expressed on T cells, a type of immune cell, which plays a crucial role in modulating T cell activation and response, making it a key player in immune responses (52, 53). The use of a combined treatment involving anti-CD6 therapy and oral insulin immunization effectively reverses recent-onset diabetes in non-obese diabetic mice (54). Alterations in CD6 levels in obesity could potentially lead to dysregulated immune responses (55), thereby contributing to the chronic inflammation associated with obesity. Previous research has shown that obesity can influence the frequency and severity of migraines (18, 56), potentially through these inflammatory pathways. The alterations in TRAIL and CD6 levels could be one of the mechanisms through which obesity influences migraine pathogenesis. However, further research is needed to fully elucidate the role of these markers in the link between obesity and migraines. Some potential causal relationship in inflammatory immunity was found for alcohol dependence, depression, anxiety disorders, insomnia, sleep apnoea, and glucocorticoid use. However, there is no evidence to support a strong colocalization relationship.

In the study, associations of circulating inflammatory proteins and immune cells with the risk of migraine were investigated. Of particular interest were the levels of T-cell surface glycoprotein CD6 isoform. Interestingly, it was found that elevated levels of this particular protein might increase the risk of migraines, with a *p*-value of less than 0.05 after FDR correction. As mentioned above, the CD6 plays a crucial role in the immune response, and alterations in its expression or function can lead to dysregulated immune activity (52, 53). In the context of migraines, it is hypothesized that elevated levels of CD6 could potentially enhance T cell activation and proliferation, contributing to the neurogenic inflammation characteristic of migraines (57, 58). However, it’s important to note that the relationship between CD6 levels and migraines is likely to be complex and multifactorial. Moreover, combined with the above analysis results, it is speculated that obesity may influence the expression of CD6, and thus affect migraine. Further studies are needed to elucidate the precise mechanisms underlying this association and to explore the potential of CD6 as a therapeutic target for migraines.

Beyond the potential impact of CD6 levels and obesity on migraines, the research unveiled connections between migraine and other three migraine risk factors, specifically, depression, all anxiety disorders, and sleep apnea. The association between all anxiety disorders and migraine was statistically significant after FDR correction. Previous researches revealed that individuals afflicted by migraines experience elevated morbidity due to recurrent headache episodes inherent to the condition, disrupted sleep patterns, and a notable prevalence of accompanying psychosocial disorders (17).

In our study, we uncovered compelling evidence indicating that depression and all anxiety disorders might heighten the risk of experiencing migraines. The simultaneous occurrence of migraines and depression poses a notable clinical conundrum, as highlighted by numerous studies (14, 29). Although conclusive evidence supporting the cause for the bidirectional link between migraine and depression is lacking, a disruption in the activation of the immune system has persistently been recognized as a significant factor (14). The pathophysiological basis of migraine is rooted in the local terminal release of products from trigeminovascular afferents, which can not only provoke the dilation of meningeal vessels but also induce a pronounced neuroinflammatory state (59). Peripheral molecular and cellular immune dysfunction impacts the central nervous system (CNS), inducing a neuroinflammatory state that is increasingly being understood in the context of depression (60). What’s more, previous study showed that anxiety exhibited a more pronounced correlation with the heightened risk of migraines compared to depression (61). The inability to effectively manage anxiety and achieve relaxation stands out as the predominant challenges in the psychiatric overlap with migraines. Physical manifestations in depression show a stronger association with migraines than emotional indications (61). In summary, our findings highlight the complex interplay between migraines, depression, and anxiety disorders. Functional changes at the molecular or cellular level in both the peripheral and central immune systems appear to be a critical pathophysiological feature that may be shared, at least in part, between migraine and depression. These results underscore the importance of a holistic approach to patient care, which takes into account not just the physical symptoms of migraines, but also the psychological factors that can exacerbate these symptoms.

Sleep apnea, characterized by repeated episodes of breathing cessation during sleep (62), was also found to significantly increase the risk of migraines in our study. However, a recent meta-analysis has found that sleep apnea does not increase the risk of headaches, which is attributed to the heterogeneity of the methods (63). Sleep apnea often results in fragmented sleep and chronic intermittent hypoxia, which may disrupt the normal sleep–wake cycle, leading to alterations in pain threshold and increased susceptibility to migraines (62). Recent studies using prospective, longitudinal methods are starting to uncover the extent and timing patterns of sleep and migraine interactions (64). Certain elements of brainstem-cortical networks associated with sleep physiology are inadvertently recognized as crucial components in the typical migraine pathway (65). Recent findings on anatomical localization, with the hypothalamus playing a pivotal early role in migraine pathophysiology, shared mediating signaling molecules like serotonin and dopamine, and the discovery of a novel CNS waste removal system, the glymphatic system, all suggest a shared pathophysiology between migraines and sleep (66).

This manuscript does not provide statistical evidence of a significant association between alcohol dependence (11, 12), smoking (13), depression (14, 15), anxiety (16), insomnia or sleep apnea (17), obesity (18), glucocorticoid use (19, 20), and migraine, though previous research has established the existence of such correlations. The heterogeneity in the results may be attributed to the use of MR methods that examine causal relationships from a genetic perspective, rather than in real-world settings. For instance, alcohol can trigger migraine attacks by causing vasodilation and stimulating pain receptors in the meninges through mechanical input (11, 12). Furthermore, alcohol consumption is a highly contentious subject, primarily due to the inherent challenge in establishing definitive guidelines for the “amounts and patterns” of its intake (67). While the focus of this study lies in alcohol dependency, future research endeavors must delve deeper into the intricacies of alcohol consumption quantities and patterns. Further research is warranted to elucidate these connections.

Our study has limitations. Firstly, our mediated MR results did not find a clear mediated effect between inflammatory proteins and immune cells in the relationship between migraine risk factors and migraine itself. This could be attributed to the limited explanatory power of genetic variations, and the intricate interactions among the three factors. Future research utilizing diverse methodologies and larger sample sizes is required to further delve into this complex relationship (14). Secondly, we primarily relay on several large-scale GWAS datasets, yet the lack of detailed demographic information hinders in-depth subgroup analyses. Thirdly, migraine varies significantly in attack frequency and disability, affecting patients differently. It can be classified as acute or chronic, but in this study, we focus specifically on acute migraine attacks, excluding chronic migraine. Furthermore, due to the inherent limitations of the MR method, our exploration of the relationships among migraine risk factors, migraine, inflammatory proteins, and immune cells is solely genetic, whereas the actual conditions are determined by a combination of environmental and genetic factors. Future studies, using different methodologies and larger sample sizes, may be able to shed more light on this complex relationship.

In summary, this study sheds new light on the potential causal links between migraine risk factors, migraine, immune cells, and inflammatory proteins. This study finds migraine risk factors linked to 276 immune cells or inflammatory proteins. And 23 immune cells and inflammatory proteins, depression, all anxiety disorders, and sleep apnea correlate with migraine. Our results do not confirm the mediating role of inflammatory proteins and immune cells in these associations. Nonetheless, this work significantly advances our understanding of the molecular etiology of migraine pathogenesis from an inflammatory-immune perspective.

## Data availability statement

The original contributions presented in the study are included in the article/Supplementary material, further inquiries can be directed to the corresponding author.

## Ethics statement

Ethical approval was not necessary for this study, as all data utilized were sourced from publicly accessible GWAS databases and did not involve any experimentation, observation, or intervention on human or animal subjects. We rigorously adhered to the data usage agreement and confidentiality requirements to safeguard the security and privacy of the data.

## Author contributions

JZ: Conceptualization, Data curation, Methodology, Software, Writing – original draft, Writing – review & editing. RC: Methodology, Supervision, Writing – review & editing. ML: Data curation, Investigation, Software, Writing – review & editing. HG: Methodology, Validation, Writing – review & editing. KL: Resources, Validation, Writing – review & editing. QZ: Funding acquisition, Project administration, Resources, Supervision, Visualization, Writing – review & editing.

## Footnotes

^1^ https://gwas.mrcieu.ac.uk/datasets/ieu-b-4826/

^2^ https://www.ebi.ac.uk/gwas/studies/GCST0001391

^3^ https://www.ebi.ac.uk/gwas/studies/GCST0002121

## References

[1] GBD 2021 Nervous System Disorders Collaborators. Global, regional, and national burden of disorders affecting the nervous system, 1990-2021: a systematic analysis for the global burden of disease study 2021. Lancet Neurol. (2024) 23:344–81. doi: 10.1016/S1474-4422(24)00038-3, PMID: 38493795 PMC10949203

[2] Ashina M Katsarava Z do TP Buse DC Pozo-Rosich P Özge A. Migraine: epidemiology and systems of care. Lancet. (2021) 397:1485–95. doi: 10.1016/S0140-6736(20)32160-7 33773613

[3] Burch RC Buse DC Lipton RB. Migraine: epidemiology, burden, and comorbidity. Neurol Clin. (2019) 37:631–49. doi: 10.1016/j.ncl.2019.06.001 31563224

[4] Safiri S Pourfathi H Eagan A Mansournia MA Khodayari MT Sullman MJM. Global, regional, and national burden of migraine in 204 countries and territories, 1990 to 2019. Pain. (2022) 163:e293–309. doi: 10.1097/j.pain.0000000000002275, PMID: 34001771

[5] Steiner TJ Terwindt GM Katsarava Z Pozo-Rosich P Gantenbein AR Roche SL. Migraine-attributed burden, impact and disability, and migraine-impacted quality of life: expert consensus on definitions from a Delphi process. Cephalalgia. (2022) 42:1387–96. doi: 10.1177/03331024221110102, PMID: 35791285 PMC9638708

[6] Ferrari MD Goadsby PJ Burstein R Kurth T Ayata C Charles A. Migraine. Nat Rev Dis Primers. (2022) 8:2. doi: 10.1038/s41572-021-00328-4 35027572

[7] Khan J Asoom LIA Sunni AA Rafique N Latif R Saif SA. Genetics, pathophysiology, diagnosis, treatment, management, and prevention of migraine. Biomed Pharmacother. (2021) 139:111557. doi: 10.1016/j.biopha.2021.111557, PMID: 34243621

[8] Grangeon L Lange KS Waliszewska-Prosół M Onan D Marschollek K Wiels W. Genetics of migraine: where are we now? J Headache Pain. (2023) 24:12. doi: 10.1186/s10194-023-01547-8, PMID: 36800925 PMC9940421

[9] Chen J Zhang S Cui K Liu C. Risk factors for benign paroxysmal positional vertigo recurrence: a systematic review and meta-analysis. J Neurol. (2021) 268:4117–27. doi: 10.1007/s00415-020-10175-0, PMID: 32839838

[10] Lipton RB Buse DC Nahas SJ Tietjen GE Martin VT Löf E. Risk factors for migraine disease progression: a narrative review for a patient-centered approach. J Neurol. (2023) 270:5692–710. doi: 10.1007/s00415-023-11880-2, PMID: 37615752 PMC10632231

[11] García-Azorín D. The complex relationship between alcohol and migraine. Headache. (2022) 62:1245–6. doi: 10.1111/head.14426, PMID: 36373782

[12] Panconesi A Bartolozzi ML Guidi L. Alcohol and migraine: what should we tell patients? Curr Pain Headache Rep. (2011) 15:177–84. doi: 10.1007/s11916-011-0184-4, PMID: 21336550

[13] Yuan S Daghlas I Larsson SC. Alcohol, coffee consumption, and smoking in relation to migraine: a bidirectional Mendelian randomization study. Pain. (2022) 163:e342–8. doi: 10.1097/j.pain.0000000000002360, PMID: 35029599

[14] Viudez-Martínez A Torregrosa AB Navarrete F García-Gutiérrez MS. Understanding the biological relationship between migraine and depression. Biomol Ther. (2024) 14:163. doi: 10.3390/biom14020163, PMID: 38397400 PMC10886628

[15] Wachowska K Bliźniewska-Kowalska K Sławek J Adamczyk-Sowa M Szulc A Maes M. Common pathomechanism of migraine and depression. Psychiatr Pol. (2023) 57:405–19. doi: 10.12740/PP/OnlineFirst/143982, PMID: 36371736

[16] Furman JM Balaban CD Jacob RG Marcus DA. Migraine-anxiety related dizziness (MARD): a new disorder? J Neurol Neurosurg Psychiatry. (2005) 76:1–8. doi: 10.1136/jnnp.2004.048926, PMID: 15607984 PMC1739317

[17] Burrowes SAB Goloubeva O Stafford K McArdle PF Goyal M Peterlin BL. Enhanced mindfulness-based stress reduction in episodic migraine-effects on sleep quality, anxiety, stress, and depression: a secondary analysis of a randomized clinical trial. Pain. (2022) 163:436–44. doi: 10.1097/j.pain.0000000000002372 34407032 PMC8669060

[18] Jahromi SR Martami F Morad Soltani K Togha M. Migraine and obesity: what is the real direction of their association? Expert Rev Neurother. (2023) 23:75–84. doi: 10.1080/14737175.2023.2173575, PMID: 36714917

[19] Tripathi RC Parapuram SK Tripathi BJ Zhong Y Chalam KV. Corticosteroids and glaucoma risk. Drugs Aging. (1999) 15:439–50. PMID: 10641955 10.2165/00002512-199915060-00004

[20] Stark RJ Stark CD. Migraine prophylaxis. Med J Aust. (2008) 189:283–8. doi: 10.5694/j.1326-5377.2008.tb02028.x 18759728

[21] Spekker E Tanaka M Szabó Á Vécsei L. Neurogenic inflammation: the participant in migraine and recent advancements in translational research. Biomedicines. (2021) 10:76. doi: 10.3390/biomedicines10010076, PMID: 35052756 PMC8773152

[22] Cacabelos R Torrellas C Fernández-Novoa L López-Muñoz F. Histamine and Immune Biomarkers in CNS Disorders. Mediat Inflamm. (2016) 2016:1924603. doi: 10.1155/2016/1924603 PMC4846752 27190492

[23] Bruno PP Carpino F Carpino G Zicari A. An overview on immune system and migraine. Eur Rev Med Pharmacol Sci. (2007) 11:245–8. PMID: 17876959

[24] Cavestro C Ferrero M Mandrino S di Tavi M Rota E. Novelty in inflammation and immunomodulation in migraine. Curr Pharm Des. (2019) 25:2919–36. doi: 10.2174/1381612825666190709204107, PMID: 31686633

[25] Chan KL Poller WC Swirski FK Russo SJ. Central regulation of stress-evoked peripheral immune responses. Nat Rev Neurosci. (2023) 24:591–604. doi: 10.1038/s41583-023-00729-2, PMID: 37626176 PMC10848316

[26] Ballesio A. Where does inflammation in insomnia come from? And does it matter for comorbidity? Sleep. (2023) 46:zsad223. doi: 10.1093/sleep/zsad223 37625028

[27] Kawai T Autieri MV Scalia R. Adipose tissue inflammation and metabolic dysfunction in obesity. Am J Physiol Cell Physiol. (2021) 320:C375–c391. doi: 10.1152/ajpcell.00379.2020, PMID: 33356944 PMC8294624

[28] Varì R Scazzocchio B Silenzi A Giovannini C Masella R. Obesity-associated inflammation: does curcumin exert a beneficial role? Nutrients. (2021) 13:1021. doi: 10.3390/nu13031021, PMID: 33809891 PMC8004232

[29] Chen SP. Migraine and treatment-resistant depression. Prog Brain Res. (2023) 281:149–73. doi: 10.1016/bs.pbr.2023.05.001 37806714

[30] Birney E. Mendelian randomization. Cold Spring Harb Perspect Med. (2022) 12:1302. doi: 10.1101/cshperspect.a041302 PMC9121891 34872952

[31] Sekula P del Greco M F Pattaro C Köttgen A. Mendelian randomization as an approach to assess causality using observational data. J Am Soc Nephrol. (2016) 27:3253–65. doi: 10.1681/ASN.2016010098, PMID: 27486138 PMC5084898

[32] Carter AR Anderson EL. Correct illustration of assumptions in Mendelian randomization. Int J Epidemiol. (2024) 53:dyae050. doi: 10.1093/ije/dyae050 38580457

[33] Sakaue S Kanai M Tanigawa Y Karjalainen J Kurki M Koshiba S. A cross-population atlas of genetic associations for 220 human phenotypes. Nat Genet. (2021) 53:1415–24. doi: 10.1038/s41588-021-00931-x, PMID: 34594039 PMC12208603

[34] Zhao JH Stacey D Eriksson N Macdonald-Dunlop E Hedman ÅK Kalnapenkis A. Genetics of circulating inflammatory proteins identifies drivers of immune-mediated disease risk and therapeutic targets. Nat Immunol. (2023) 24:1540–51. doi: 10.1038/s41590-023-01588-w, PMID: 37563310 PMC10457199

[35] Orrù V Steri M Sidore C Marongiu M Serra V Olla S. Complex genetic signatures in immune cells underlie autoimmunity and inform therapy. Nat Genet. (2020) 52:1036–45. doi: 10.1038/s41588-020-0684-4, PMID: 32929287 PMC8517961

[36] Burgess S Thompson SG. Avoiding bias from weak instruments in Mendelian randomization studies. Int J Epidemiol. (2011) 40:755–64. doi: 10.1093/ije/dyr036, PMID: 21414999

[37] Pierce BL Burgess S. Efficient design for Mendelian randomization studies: subsample and 2-sample instrumental variable estimators. Am J Epidemiol. (2013) 178:1177–84. doi: 10.1093/aje/kwt084, PMID: 23863760 PMC3783091

[38] Bowden J Davey Smith G Burgess S. Mendelian randomization with invalid instruments: effect estimation and bias detection through egger regression. Int J Epidemiol. (2015) 44:512–25. doi: 10.1093/ije/dyv080, PMID: 26050253 PMC4469799

[39] Greco M FD Minelli C Sheehan NA Thompson JR. Detecting pleiotropy in Mendelian randomisation studies with summary data and a continuous outcome. Stat Med. (2015) 34:2926–40. doi: 10.1002/sim.6522, PMID: 25950993

[40] Bowden J Davey Smith G Haycock PC Burgess S. Consistent estimation in Mendelian randomization with some invalid instruments using a weighted median estimator. Genet Epidemiol. (2016) 40:304–14. doi: 10.1002/gepi.21965, PMID: 27061298 PMC4849733

[41] Verbanck M Chen CY Neale B do R. Detection of widespread horizontal pleiotropy in causal relationships inferred from Mendelian randomization between complex traits and diseases. Nat Genet. (2018) 50:693–8. doi: 10.1038/s41588-018-0099-7, PMID: 29686387 PMC6083837

[42] Hemani G Tilling K Davey Smith G. Orienting the causal relationship between imprecisely measured traits using GWAS summary data. PLoS Genet. (2017) 13:e1007081. doi: 10.1371/journal.pgen.1007081, PMID: 29149188 PMC5711033

[43] Glickman ME Rao SR Schultz MR. False discovery rate control is a recommended alternative to Bonferroni-type adjustments in health studies. J Clin Epidemiol. (2014) 67:850–7. doi: 10.1016/j.jclinepi.2014.03.012, PMID: 24831050

[44] Zuber V Grinberg NF Gill D Manipur I Slob EAW Patel A. Combining evidence from Mendelian randomization and colocalization: review and comparison of approaches. Am J Hum Genet. (2022) 109:767–82. doi: 10.1016/j.ajhg.2022.04.001, PMID: 35452592 PMC7612737

[45] Milad N Pineault M Lechasseur A Routhier J Beaulieu MJ Aubin S. Neutrophils and IL-1α regulate surfactant homeostasis during cigarette smoking. J Immunol. (2021) 206:1923–31. doi: 10.4049/jimmunol.2001182, PMID: 33722877

[46] Rainero I Pinessi L Salani G Valfrè W Rivoiro C Savi L. A polymorphism in the interleukin-1alpha gene influences the clinical features of migraine. Headache. (2002) 42:337–40. doi: 10.1046/j.1526-4610.2002.02103.x, PMID: 12047332

[47] Uekita T Sakai R. Roles of CUB domain-containing protein 1 signaling in cancer invasion and metastasis. Cancer Sci. (2011) 102:1943–8. doi: 10.1111/j.1349-7006.2011.02052.x, PMID: 21812858

[48] Music M Soosaipillai A Batruch I Prassas I Bogdanos DP Diamandis EP. A proteome-wide immuno-mass spectrometric identification of serum autoantibodies. Clin Proteomics. (2019) 16:25. doi: 10.1186/s12014-019-9246-0 31249498 PMC6585069

[49] Yılmaz IA Özge A Erdal ME Edgünlü TG Çakmak SE Yalın OÖ. Cytokine polymorphism in patients with migraine: some suggestive clues of migraine and inflammation. Pain Med. (2010) 11:492–7. doi: 10.1111/j.1526-4637.2009.00791.x, PMID: 20113413

[50] Keuper M Wernstedt Asterholm I Scherer PE Westhoff MA Möller P Debatin KM. TRAIL (TNF-related apoptosis-inducing ligand) regulates adipocyte metabolism by caspase-mediated cleavage of PPARgamma. Cell Death Dis. (2013) 4:e474. doi: 10.1038/cddis.2012.212, PMID: 23348588 PMC3563999

[51] Funcke JB Zoller V Hay MAE Debatin KM Wabitsch M Fischer-Posovszky P. TNF-related apoptosis-inducing ligand promotes human preadipocyte proliferation via ERK1/2 activation. FASEB J. (2015) 29:3065–75. doi: 10.1096/fj.14-267278, PMID: 25857555 PMC4478800

[52] Gonçalves CM Henriques SN Santos RF Carmo AM. CD6, a rheostat-type signalosome that Tunes T cell activation. Front Immunol. (2018) 9:2994. doi: 10.3389/fimmu.2018.02994, PMID: 30619347 PMC6305463

[53] Henriques SN Oliveira L Santos RF Carmo AM. CD6-mediated inhibition of T cell activation via modulation of Ras. Cell Commun Signal. (2022) 20:184. doi: 10.1186/s12964-022-00998-x, PMID: 36414966 PMC9682754

[54] Schneider DA Sarikonda G Montero E von Herrath M. Combination therapy with anti-CD6 and oral insulin immunization reverses recent onset diabetes in non obese diabetic mice but fails to induce lasting tolerance. Clin Immunol. (2013) 149:440–1. doi: 10.1016/j.clim.2013.08.004, PMID: 24211845

[55] Chalmers SA Ayilam Ramachandran R Garcia SJ der E Herlitz L Ampudia J. The CD6/ALCAM pathway promotes lupus nephritis via T cell-mediated responses. J Clin Invest. (2022) 132:825–35. doi: 10.1172/JCI147334, PMID: 34981775 PMC8718154

[56] Fortini I Felsenfeld Junior BD. Headaches and obesity. Arq Neuropsiquiatr. (2022) 80:204–13. doi: 10.1590/0004-282x-anp-2022-s106, PMID: 35976296 PMC9491411

[57] Faraji F Shojapour M Farahani I Ganji A Mosayebi G. Reduced regulatory T lymphocytes in migraine patients. Neurol Res. (2021) 43:677–82. doi: 10.1080/01616412.2021.1915077, PMID: 33853506

[58] Thonnard-Neumann E Neckers LM. T-lymphocytes in migraine. Ann Allergy. (1981) 47:325–7. PMID: 6976139

[59] Kursun O Yemisci M van den Maagdenberg AMJM Karatas H. Migraine and neuroinflammation: the inflammasome perspective. J Headache Pain. (2021) 22:55. doi: 10.1186/s10194-021-01271-1 34112082 PMC8192049

[60] Nettis MA Pariante CM. Is there neuroinflammation in depression? Understanding the link between the brain and the peripheral immune system in depression. Int Rev Neurobiol. (2020) 152:23–40. doi: 10.1016/bs.irn.2019.12.004, PMID: 32450998

[61] Peres MFP Mercante JPP Tobo PR Kamei H Bigal ME. Anxiety and depression symptoms and migraine: a symptom-based approach research. J Headache Pain. (2017) 18:37. doi: 10.1186/s10194-017-0742-1, PMID: 28324317 PMC5360747

[62] Tiseo C Vacca A Felbush A Filimonova T Gai A Glazyrina T. Migraine and sleep disorders: a systematic review. J Headache Pain. (2020) 21:126. doi: 10.1186/s10194-020-01192-5, PMID: 33109076 PMC7590682

[63] Błaszczyk B Martynowicz H Więckiewicz M Straburzyński M Antolak M Budrewicz S. Prevalence of headaches and their relationship with obstructive sleep apnea (OSA) – systematic review and meta-analysis. Sleep Med Rev. (2024) 73:101889. doi: 10.1016/j.smrv.2023.101889, PMID: 38056382

[64] Vgontzas A Pavlović J Bertisch S. Sleep symptoms and disorders in episodic migraine: assessment and management. Curr Pain Headache Rep. (2023) 27:511–20. doi: 10.1007/s11916-023-01160-z, PMID: 37665530

[65] Vgontzas A Pavlović JM. Sleep disorders and migraine: review of literature and potential pathophysiology mechanisms. Headache. (2018) 58:1030–9. doi: 10.1111/head.13358, PMID: 30091160 PMC6527324

[66] Song TJ Kim BS Chu MK. Therapeutic role of melatonin in migraine prophylaxis: is there a link between sleep and migraine? Prog Brain Res. (2020) 255:343–69. doi: 10.1016/bs.pbr.2020.05.014 33008513

[67] Błaszczyk B Straburzyński M Więckiewicz M Budrewicz S Niemiec P Staszkiewicz M. Relationship between alcohol and primary headaches: a systematic review and meta-analysis. J Headache Pain. (2023) 24:116. doi: 10.1186/s10194-023-01653-7, PMID: 37612595 PMC10463699

